# Case Report: Microglia Composition and Immune Response in an Immunocompetent Patient With an Intracranial Syphilitic Gumma

**DOI:** 10.3389/fneur.2020.615434

**Published:** 2021-01-13

**Authors:** Qian Yu, Wei Li, Xiaohui Mo, Fei Tan, Lianjuan Yang

**Affiliations:** ^1^Department of Medical Mycology, Shanghai Dermatology Hospital, Shanghai, China; ^2^Department of Medical Cosmetology, Shanghai Dermatology Hospital, Shanghai, China; ^3^Department of Dermatology, Renji Hospital, School of Medicine, Shanghai Jiaotong University, Shanghai, China; ^4^Department of Science and Education, Shanghai Dermatology Hospital, Shanghai, China

**Keywords:** M2 microglia, humoral immunity, *Treponema pallidum*, microglia, intracranial syphilitic gumma

## Abstract

The pathogenesis of intracranial syphilitic gummas remains poorly understood. Microglia are generally considered to be the main cell type of the innate immune system in the brain. Determination of the composition of infiltrating microglia of patients with typical intracranial syphilitic gummas may contribute to the understanding of the pathological process. We report a case of an intracranial syphilitic gumma who presented with right upper limb weakness. The histological analysis showed the presence of *Treponema pallidum* and infiltration with histiocytes. Immunostaining indicated that cells were predominantly the M2a and M2c, which were Arg-1^+^ and IL-10^+^. These findings suggest that there is an increased number of M2a/M2c microglia in intracranial syphilitic gummas, which may be part of the immune escape mechanisms triggered by *Treponema pallidum*.

## Introduction

Tertiary syphilis, which can present as an intracranial syphilitic gumma, is a complication of long-term infection with *Treponema pallidum*, which develops 1–46 years after the healing of secondary lesions; most cases develop within 15 years (1). It is frequently misdiagnosed as other intracranial space occupying lesions such as gliomas, glioblastomas, or metastatic tumors. Pathologically, syphilitic gummas are thick, tough, rubbery lesions of fibrous trabecula with lymphocytic and plasma cell infiltrations of the outer layers (2).

The pathogenesis of intracranial syphilitic gummas remains poorly understood. Cumulative evidence has demonstrated that both innate immune and adaptive immunities contribute to the pathological damage in the central nervous system (CNS) in neurosyphilis. Microglia (3), the resident macrophage cells of the CNS, is generally considered the main cell type of the innate immune system in the brain. Determination of the composition of infiltrating microglia and immune response status of patients with typical intracranial syphilitic gummas may contribute to the understanding of the pathological process involved in this pathology.

We report a case of an intracranial syphilitic gumma resembling a malignant brain tumor and analyze the composition of microglia in the patient's brain. The patient signed a written informed consent form, and the study was approved by the ethics committee of Shanghai Dermatology Hospital.

## Case Presentation

A 54-year-old heterosexual male patient presented with a 10-day history of right upper limb weakness. He had no history of previous diseases. At admission, the patient had decreased muscle force (three grades) and decreased muscle tone in the right upper limb. The remaining neurological examination was unremarkable. There was no other significant medical history and no family of neuromuscular disease. The patient denied any history of suggestive of syphilis. He had irregular extramarital sexual behavior since ten years ago and never received formal antisyphilitic treatment.

**Figure 1:**
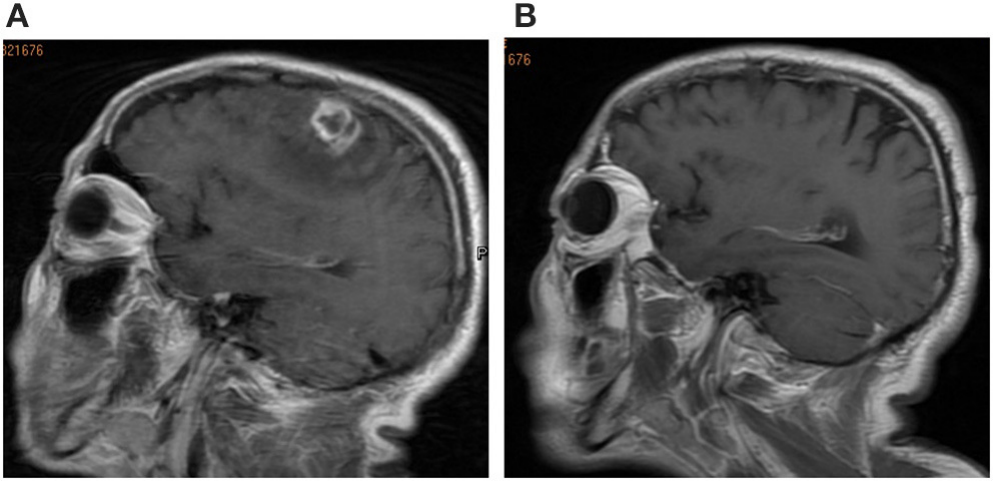
**(A)** Brain magnetic resonance imaging (MRI) revealed an irregular enhancing mass with central necrosis on the left parietal lobe. **(B)** Brain MRI image after surgery at the follow-up 3-mouth.

Hematological investigation revealed a leukocyte count of 8.18 × 10^9^/L. Blood, renal, liver tests, immunological tests, tumor markers, peripheral blood lymphocyte subsets, immunoglobulins, and complements were all within normal limits. Computed tomography (CT) scans of the chest and abdomen were normal. Brain CT showed a space-occupying lesion in the left frontal lobe. Subsequent sagittal magnetic resonance imaging (MRI) revealed an irregular enhancing mass with central necrosis on the left parietal lobe that measured 15 × 13 × 10 mm in size (Figure 1A). There was extensive cerebral edema around the enhancing mass. The radiological appearance was suggestive of a meningioma or metastatic tumor.

**Figure 2:**
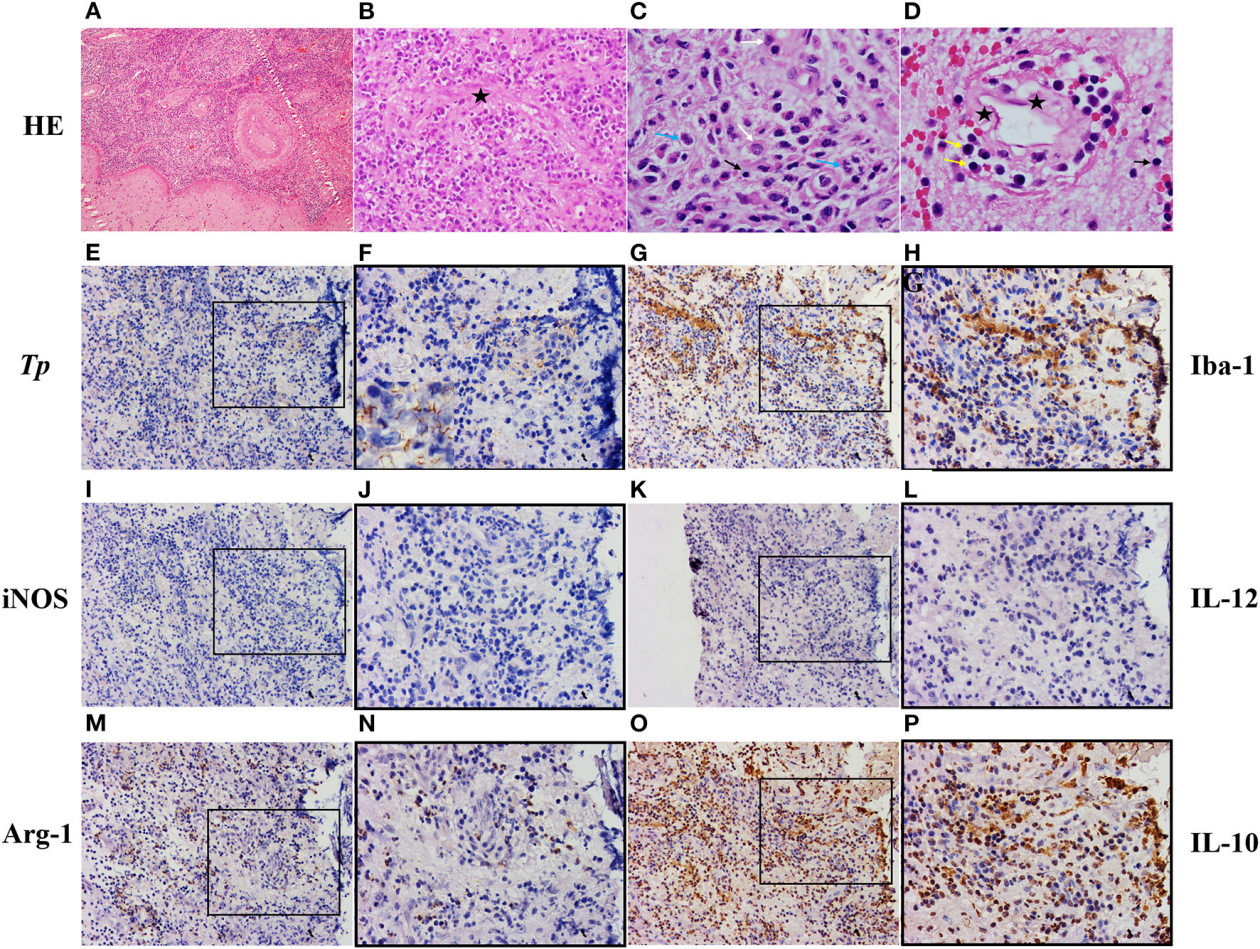
Pathologic diagnosis of the intracranial syphilitic gumma and cellular organization of microglia. **(A–D)** Hematoxylin and eosin staining of the intracranial syphilitic gumma reveals that the central portion of the mass contains a necrotizing inflammatory material infiltrated with epithelioid cells(white arrows), lymphocytes(black arrows), plasma cells(yellow arrows), and neutrophils(blue arrows). The vessels show severe endarteritis with endothelial cell swelling (black asterisks) and hyperplasia. **(E,F)** Immunostaining showing spirochetes in the intracranial syphilitic gumma. **(G,H)** Immunostaining for Iba-1 in the intracranial syphilitic gumma. **(I,J)** Immunostaining for iNOS in the intracranial syphilitic gumma. **(K,L)** Immunostaining for IL-12 in the intracranial syphilitic gumma. **(M,N)** Immunostaining for Arg in the intracranial syphilitic gumma. **(O,P)** Immunostaining for IL-10 in the intracranial syphilitic gumma. F, H, J, L, N, P are partial magnifications of E, G, I, K, M, O, respectively. Original magnifications: A, (×10); E, G, I, M, O, (×20); B, F, H, J, L, N, P, (×40); C, D, (×100).

The patient underwent a left temporal craniotomy for excision of the lesion under general anesthesia. Postoperative pathological analysis of the resected tissue showed that the central portion of the mass contained a necrotizing inflammatory material(Figures 2A,B) infiltrated with epithelioid cells(Figure 2C), lymphocytes(Figures 2C,D), plasma cells(Figure 2D), and neutrophils(Figure 2C). The blood vessels showed severe endarteritis with endothelial cell swelling and hyperplasia (Figures 2B,D). These findings were consistent with a chronic inflammatory granulomatous process.

Syphilis serology was strongly positive for both treponemal and non-treponemal specific antibodies with a rapid-plasma regain (RPR) titer of 1:64. The patient's HIV test result was negative. Spirochete immunostaining (Figures 2E,F) highlighted many organisms, typical spiral and thread-like, in the excised lesion. Eventually, the patient was diagnosed with an intracranial syphilitic gumma and treated with aqueous crystalline penicillin G, 4 million units intravenously every 4 hours for 14 days. At the 3-month follow-up, an 8-fold decrease in the RPR titer was observed. The brain MRI scans of the patient showed no enhancing mass lesion or edema in the cerebral parenchyma (Figure 1B). The patient is presently undergoing serological follow-up, and his serum RPR titer has stabilized at 1:4 after 6 months of treatment.

Treponemes have seldom been demonstrated in the gummas in existing literature (2, 4, 5). In this case, immunohistochemical staining revealed numerous spirochetes. Immunostaining for *T. pallidum* also showed many histiocytes surrounded by treponemes and rare lymphocytes surrounded by treponemes (Figures 2E,F). Therefore, we explored the cell composition around the *T. pallidum* and performed immunostaining on the serial cerebral sections. We observed that many histiocytes were gathered around the *T. pallidum*, and these cells expressed ionized calcium-binding adapter molecule 1 (Iba-1) (Figures 2G,H), which is specifically expressed in activated microglia. By counting the number of positive cells and the total number of cells in the gumma using Aperio ImageScope, we observed that the percentage of Iba-1^+^ microglia in total cells was 30% (Figures 2G,H). We further investigated the heterogeneity and biological properties of microglia in this case of an intracranial syphilitic gumma. We found that a 26% subset of Iba-1^+^ microglia is Arg-1^+^ microglia (M2-phenotype) (Figures 2M,N), and iNOS^+^ microglia (M1-phenotype) (Figures 2I,J) was negative. In addition, M1 microglia-associated cytokine IL-12 showed negative expression (Figures 2K,L), the level of CSF IL-12p70 was 0.24pg/ml. M2 microglia-associated cytokine IL-10 indicated diffuse high expression (Figures 2O,P), the level of CSF IL-10 was 4.12pg/ml.

## Discussion

Chronic smoldering inflammation in intracranial syphilitic gummas is reflective of a persistent immune response to *T. pallidum*, and/or its residual antigens, in patients unable to mount a completely effective delayed-type hypersensitivity (DTH) response (6). As in many other non-bacterial infectious diseases, including viral and mycobacterial infections, DTH responses have been shown to be responsible for curing infections, whereas humoral immunity responses are associated with prolonged progressive infections (6). In a previous study, we verified that ectopic germinal centers, which are important structures for the maintenance of humoral immunity, exist in intracranial syphilitic gummas (7). Aberrant humoral immune responses could represent an important step in disease exacerbation. This might explain why patients with syphilis and HIV co-infection tend to have accelerated emergence of tertiary symptoms as well as increased risk of developing neurological complications, through impaired cellular immunity and compensatory activated humoral immunity.

Surprisingly, we noted that *T. pallidum* was distributed mainly around the histiocytes and scarcely around the lymphocytes. Image analysis showed that these histiocytes express Iba-1, which is up-regulated in microglia following CNS nerve injury (8) in ischemia, and several other brain diseases. Microglia activation and proliferation occur in almost all pathologies affecting the CNS. “Classical” activation (M1) is known to play a pro-inflammatory role to exacerbate tissue damage; anti-inflammatory states (M2) have been implicated in tissue repair, matrix deposition, and resolution of pro-inflammatory. The latter further classified into three subsets: M2a (elicited by IL-4 and IL-13), which promotes typeII immune responses and fibrogenesis, M2b (stimulated by immune complexes), which is immunoregulatory, and M2c (stimulated by IL-10, TGF-β or glucocorticoids) which is anti-inflammatory and initiates tissue remodeling (9, 10). The previous studies indicated that the level of cerebrospinal fluid (CSF) IL-4, IL-10, and IL-13 in patients with neurosyphilis was significantly higher than that in those who did not have neurosyphilis. However, no significant differences were found in CSF IL-12p70 between those with and without neurosyphilis (Supplementary Figure 1, unpublished data). This indirectly suggests an increased number of M2a/M2c microglia in the CNS of patients with neurosyphilis.

Both of M2a and M2c can secrete IL-10, which inhibits production of pro-inflammatory cytokines such as TNF-α, IL-6, IL-12, and antigen presentation by monocytes or macrophage via downregulation of MHCII and costimulatory molecules (11). Some bacterial pathogens, such as *Brucella abortus* (12), *Mycobacterium tuberculosis, Mycobacterium leprae* (13), *Coxiella burnetii* (14), and *Tropheryma whipplei* (15), have been shown to suppress innate host defense mechanisms by shifting monocyte polarization toward an M2a profile associated with IL-10 secretion, and preventing M1 polarization and IL-12 production. In our case, plenty of Arg1^+^ M2 microglial cells were found around the *T. pallidum* and displayed co-localization with IL-10^+^ cells. We speculate that these characteristic M2 microglia associated with IL-10 might promote disease progression possibly by creating an immunosuppressive microenvironment in intracranial syphilitic gummas. *T. pallidum* can then survive, protected from killing by immune cells, and persist in a latent or progressive form. M2a/M2c microglia may be part of the immune escape mechanisms triggered by *T. pallidum*.

There were some limitations in this study. First, only one case was rare entity. However, we do not have other samples due to the rarity of intracranial syphilitic gumma. In addition, studies of macrophage polarization and function are needed to further explore, but these experiments have been hampered by inherent difficulty in conducting immunologic studies of neurosyphilis in experimental animal models.

As the contribution of immune processes in the etiology of intracranial syphilitic gummas is still not fully known, we analyzed the microglia composition and immune status in a typical case, which may provide novel clues to understanding the pathogenesis of this disease. Future therapeutic strategies for disease treatment in individuals with neurosyphilis should focus on accelerating the transition of the CNS immune response from an anti- to a pro-inflammatory status.

## Informed Consent

Informed consent was obtained from the participant included in this study.

## Data Availability Statement

The original contributions presented in the study are included in the article/Supplementary Materials, further inquiries can be directed to the corresponding author/s.

## Ethics Statement

The studies involving human participants were reviewed and approved by this study involving human participant was reviewed and approved by the Ethics Committee of Shanghai Dermatology Hospital. The patient provided his written informed consent to participate in this study. The patients/participants provided their written informed consent to participate in this study. Written informed consent was obtained from the individual(s) for the publication of any potentially identifiable images or data included in this article.

## Author Contributions

QY and WL: design and draft of the work, analysis, acquisition, and interpretation of data. XM: laboratory testing and data analysis. FT and LY: study design, revised, and finalized the manuscript. All authors contributed to the article and approved the submitted version.

## Conflict of Interest

The authors declare that the research was conducted in the absence of any commercial or financial relationships that could be construed as a potential conflict of interest.

## Supplementary Material

Supplementary Figure 1 **(A)** The level of CSF IL-4 was significantly higher in neurosyphilis patients than those in non-neurosyphilis patients. **(B)** The level of CSF IL-13 was significantly higher in neurosyphilis patients than those in non-neurosyphilis patients. **(C)** The level of CSF IL-10 was significantly higher in neurosyphilis patients than those in non-neurosyphilis patients. **(D)** The level of CSF-IL-12p70 was no significant difference between neurosyphilis and non-neurosyphilis patients.
