# Supplementary figures and images for: Case Report: Microglia Composition and Immune Response in an Immunocompetent Patient With an Intracranial Syphilitic Gumma
Source: Front Neurol. 2021 Jan 13;11:615434. doi: 10.3389/fneur.2020.615434 (PMC7838610; doi:10.3389/fneur.2020.615434)

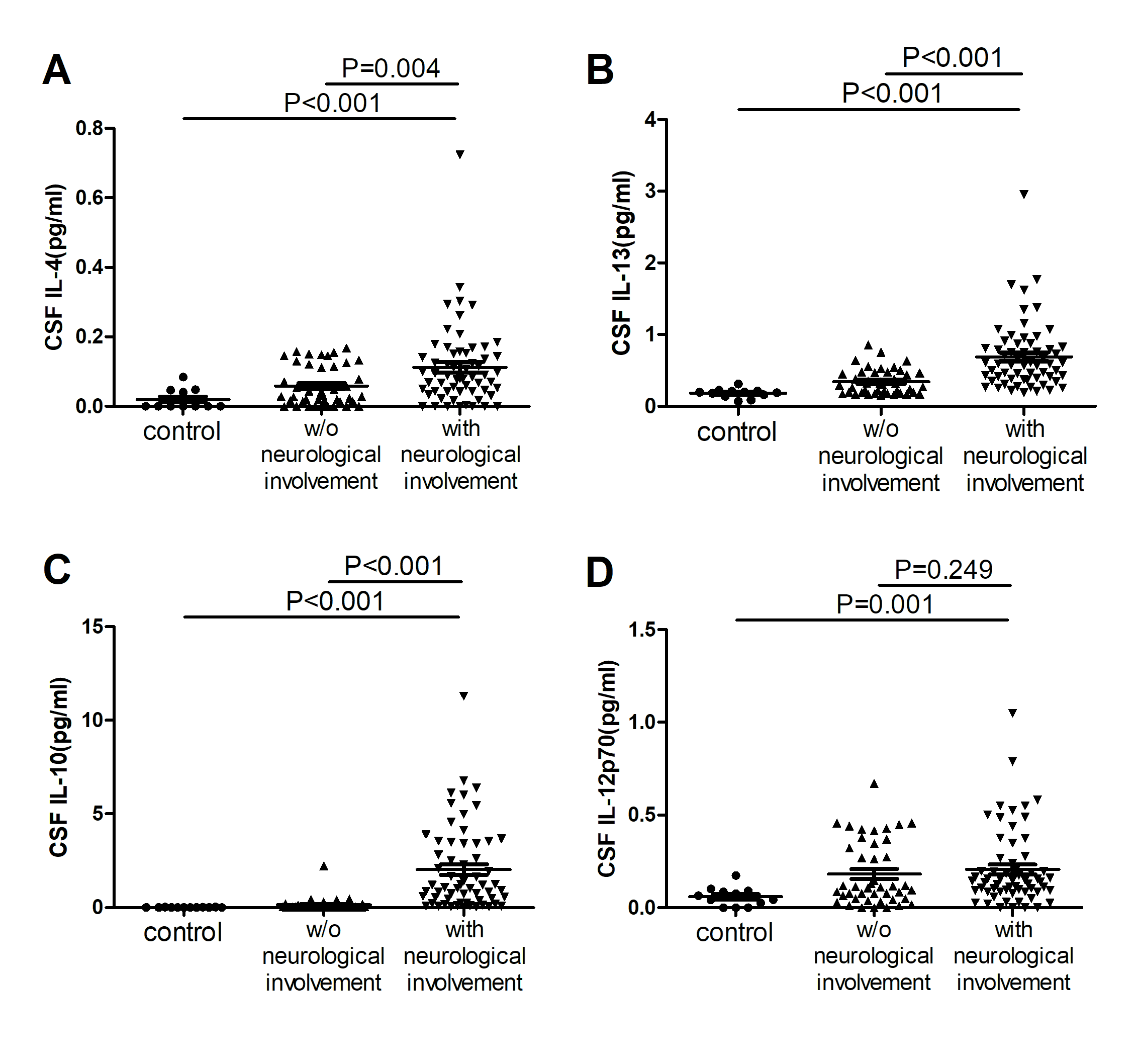

Supplement: Supplementary Figure 1 — (A) The level of CSF IL-4 was significantly higher in neurosyphilis patients than those in non-neurosyphilis patients. (B) The level of CSF IL-13 was significantly higher in neurosyphilis patients than those in non-neurosyphilis patients. (C) The level of CSF IL-10 was significantly higher in neurosyphilis patients than those in non-neurosyphilis patients. (D) The level of CSF-IL-12p70 was no significant difference between neurosyphilis and non-neurosyphilis patients. [file Image_1.TIF]
